# The butterfly effect in clinical supervision

**DOI:** 10.1007/s40037-021-00659-8

**Published:** 2021-03-22

**Authors:** Jennifer M. Klasen, Lorelei A. Lingard

**Affiliations:** 1 Clarunis, Department of Visceral Surgery, University Center for Gastrointestinal and Liver Diseases, St. Claraspital and University Hospital Basel, Basel, Switzerland; 2 Centre for Education Research and Innovation, Schulich School of Medicine and Dentistry, Western University, London, Ontario Canada; 3 Department of Medicine, University of Western Ontario, London, Ontario Canada

## “What does the flap of a butterfly’s wing look like in clinical supervision?”

The butterfly effect is a well-known metaphor for the idea that complex, dynamic, nonlinear systems produce unpredictable effects due to the profound influence of tiny variations [1]. There are tacit assumptions of linearity made about many things in medical education, such as the way we talk about the progression of a trainee from incompetence to competence or the graded autonomy supervisors allow trainees over the course of this progression [2–6]. However, we also recognize that linear assumptions don’t always hold in our field [7]. To understand complex work-based training phenomena like supervision and entrustment, we need to acknowledge that the interactions among clinicians, trainees and patients are nonlinear [7]. This intersection, this tension between linear and nonlinear assumptions, is a tricky space to inhabit as educators and as researchers. But we must, if we are to develop robust understandings of how supervision and entrustment work in practice.

Gilchrist et al.’s multiple case study of supervisory dyads offers us a glimpse of this intersection [8]. The group explored how supervisory behaviours related to their judgments of trainee competence. They acknowledge that clinical supervision is a “dynamic activity”, and that “what appears to be a linear path towards an entrustment decision, may actually represent a complex interaction of factors” [8]. At the same time, they conceptualize the activity under study in a linear manner: a trigger produces a supervisory behaviour that shapes a competence judgment which impacts future supervisory behaviour. This linear conceptualization is reflected in the systematic approach by which the analysis sought “to organize the information pertaining to each incident by parsing it into information that described the supervisory behaviour, the trigger of the supervisory behaviour, why the attending responded to that particular situation with that particular supervisory behaviour, how the incident informed their judgment of trainee competence, and any impact on subsequent supervision” [8].

We are not arguing against this analytical approach. There is an elegant logic to it. The study offers an impressive dataset of 10 cases, 51 interview transcripts and 25 sets of daily field notes, which yielded 1–7 supervisory incidents per case for a total of 37 incidents within each case. A rich description of trainee and nontrainee triggers, supervisory behaviours and competence judgments helps to advance our understanding of supervisory practices in the clinical workplace. But, perhaps a bit ironically, one of the main findings is that there is “not a consistent relationship between the trigger for supervision, the supervisor’s competence judgment of the trainee, and the supervisory behaviour, both within the (presented) dyads and across dyads” in the study [8].

It may be that we feel this irony because we’ve inhabited this same space as researchers. Our research explores the supervisory strategy of allowing failure in clinical training, asking supervisors about situations in which they allowed trainees to fail for educational purposes [9]. Supervisors reported that their decisions to allow failure were intuitive, made in the moment and perhaps even unconscious. Reflecting on these decisions afterwards, they realized a complex set of patient, trainees, supervisor and environmental factors interacting to produce these intuitive decisions. These factors sounded linear, particularly the recurring notion that “patient factors trump all”. But when we tried to model the relationships between the factors as a way of understanding why failure might be allowed in one situation but not another, we concluded that the answer was “it depends”. Even patient factors were not straightforwardly linear—that is, they did not predict the decision to allow failure for learning—because they worked in combination with the other factors. Again, we come up against the tension between linearity and nonlinearity. Clinical supervisors decide to allow failure in one moment and they describe factors that explain the decision afterwards, but those factors do not seem to predict their next decision. Rather, they may decide not to allow failure the next time, even when the factors appear similar on the surface. Something has changed in the interplay of patient, trainee, supervisor and environment factors, beneath the surface of their awareness and our view as researchers. Because it is something we can’t predict or articulate, it manifests itself as “it depends” in our dataset.

We are not alone in recognizing such complexity in medical education research. In fact, nonlinearity is a recurring finding from our community. We may not always be using this term, but that’s what we’re bumping up against. It may appear in the literature as “it depends” research [7]. For instance, Ginsberg et al. used focus groups to explore practicing physicians’ approaches to common professionalism dilemmas and found that, although participants agreed on basic guiding principles of professionalism, their reported approaches “were subject to multiple, interdependent, idiosyncratic forces unique to each situation”, making their responses “difficult to predict or assess” (p. 1692) [10]. Titling their paper “It depends: …”, they concluded that professionalism should be approached as “a complex adaptive system … in which multiple interdependent factors operate simultaneously” such that even the few rules that appeared to govern responses in one situation may be broken in another (p. 1692).

Even as our models of clinical supervision become increasingly sophisticated, we run up against this “it depends” problem. Take two recent examples. Hauer et al.’s phenomenographic study (2015) of how supervisors judge a resident’s trustworthiness for practice identified accelerators and barriers that interact to influence the evolution of trust formation [11]. And Holzhausen’s (2017) conceptual framework of the entrustment decision-making process combined factors identified through empirical research in medical education with theoretical models on trust from the fields of organizational and occupational psychology, in order to support research into the rich array of variables influencing the entrustment decision-making process [12]. In both of these works, we see the crossroads of linearity and nonlinearity as researchers grapple with complex, dynamic processes. Holzhausen et al. identify “potentially important variables and their interrelatedness, with the goal of making these assumptions explicit and testable” (p. 123), while at the same time acknowledging that “it is not yet clear how strong the effects of various factors are”. In addition, there remain a number of “unknown influential variables in the entrustment decision making process” including “subconscious factors within the trustor”, “mood”, and “gut feeling” (p. 124). Similarly, Hauer et al. acknowledged that the process of developing trust is “complex and sometimes nebulous” (p. 792) and they warn that it “can involve a synthetic, holistic judgement that perhaps cannot be fragmented into milestones” (p. 792). Yet, their conclusion sits at the very intersection of linearity and nonlinearity, both emphasizing “the complexity and dynamically evolving nature of trust” and suggesting that “the development of trust could be standardised using trust-based ratings scales” (p. 793) [11].

Scholars exploring the dynamic processes of clinical supervision and entrustment will perhaps always look up from their work and find themselves in the land of “it depends”, between the proverbial rock (of linearity) and the hard place (of nonlinearity). What do we do with this? Let’s return to the butterfly’s wing, that famous icon of chaos theory. Back in 1972, a professor at MIT asked, “*Does the flap of a butterfly’s wings in Brazil set off a tornado in Texas?”* (from Edward U. Lorenz, Professor of Meteorology, Massachusetts Institute of Technology, Cambridge, 1972). The butterfly’s wing is a trigger, but not in the way that Gilchrist et al. conceptualize. In their work, a trigger is a visible or audible cue to which the supervisor is observed to respond in a linear fashion. The butterfly’s wing, by contrast, is an invisible, inaudible trigger: it happens in Brazil, so the tornado victims in Texas *cannot* respond. We would encourage extending Gilchrist et al.’s trigger concept to include nonlinear triggers—to include butterfly’s wings. This conceptualization might help us to explore supervisory responses for which there is no visible or audible cue. How do we understand those responses? Are they triggerless? Or are supervisors responding to invisible, inaudible cues? And if they are, are there ways for us to render those cues visible and audible—to supervisors, and to researchers?

Such questions could help us to push ourselves to deepen our exploration at the crossroads of linearity and nonlinearity. Not least, they could position us to explore the implications of nonlinearity, of “it depends” phenomenon, for both trainee learning and patient safety. If a mere flap of a butterfly’s wing can change the nature of clinical supervision, then how can we guarantee optimal trainee learning and patient safety? We cannot, unless our research advances to make these small disturbances recognizable and provide a new language for talking about them. What appears as chaos, as unpredictable in any single study, may present itself as an emergent pattern if we can step back and take a wider view. We should not, however, expect that pattern to be linear. Nonlinearity may be uncomfortable for us, but we must challenge ourselves to describe these dynamic phenomena without slipping into linear assumptions.
